# Plant Sterols and Plant Stanols in Cholesterol Management and Cardiovascular Prevention

**DOI:** 10.3390/nu15132845

**Published:** 2023-06-22

**Authors:** Fotios Barkas, Eirini Bathrellou, Tzortzis Nomikos, Demosthenes Panagiotakos, Evangelos Liberopoulos, Meropi D. Kontogianni

**Affiliations:** 1 Department of Hygiene & Epidemiology, Faculty of Medicine, School of Health Sciences, University of Ioannina, 45110 Ioannina, Greece; 2 Department of Nutrition and Dietetics, School of Health Sciences and Education, Harokopio University, 17676 Kallithea, Greece; 3 1st Propaedeutic Department of Medicine, General Hospital of Atherns ‘Laiko’, School of Medicine, National and Kapodistrιan University of Athens, 11527 Athens, Greece

**Keywords:** plant sterols, plant stanols, cardiovascular disease, cholesterol, supplements, functional foods

## Abstract

Atherosclerotic cardiovascular disease (ASCVD) remains the major mortality cause in developed countries with hypercholesterolaemia being one of the primary modifiable causes. Lifestyle intervention constitutes the first step in cholesterol management and includes dietary modifications along with the use of functional foods and supplements. Functional foods enriched with plant sterols/stanols have become the most widely used nonprescription cholesterol-lowering approach, despite the lack of randomized trials investigating their long-term safety and cardiovascular efficacy. The cholesterol-lowering effect of plant-sterol supplementation is well-established and a potential beneficial impact on other lipoproteins and glucose homeostasis has been described. Nevertheless, experimental and human observational studies investigating the association of phytosterol supplementation or circulating plant sterols with various markers of atherosclerosis and ASCVD events have demonstrated controversial results. Compelling evidence from recent genetic studies have also linked elevated plasma concentrations of circulating plant sterols with ASCVD presence, thus raising concerns about the safety of phytosterol supplementation. Thus, the aim of this review is to provide up-to-date data on the effect of plant sterols/stanols on lipid-modification and cardiovascular outcomes, as well as to discuss any safety issues and practical concerns.

## 1. Introduction

Cardiovascular disease (CVD) is the main cause of mortality and increased socioeconomic costs in developed countries [1]. It is estimated that four million deaths are caused by CVD in Europe, which corresponds to 45% of annual deaths [1]. CVD costs the European Union economy €210 billion, of which 53% (€111 billion) is due to healthcare costs, 26% (€54 billion) to productivity losses, and 21% (€45 billion) to the informal care of people with CVD [1]. Among other risk factors such as obesity, diabetes, metabolic syndrome, smoking and hypertension, dyslipidaemia remains the primary cause of CVD [2]. Elevated levels of cholesterol, and especially low-density lipoprotein cholesterol (LDL-C), have been associated with an increased incidence of coronary artery disease (CAD) [2]. Based on this evidence, therapeutic interventions aiming at LDL-C reduction in the setting of primary and secondary cardiovascular prevention have been developed [2]. Indeed, a few meta-analyses have confirmed that any LDL-C lowering intervention, either dietary or pharmaceutical, effectively reduces CVD risk [3].

Dietary interventions are the initial step in all international guidelines for the management of dyslipidaemias and cardiovascular prevention as they can reduce LDL-C levels by ~20% and CVD risk [2]. Since the publication of the seven-country cohort, many studies have investigated the association of individual food components or dietary patterns with cardiovascular risk factors and CVD incidence [4]. In addition to dietary modifications, supplements and functional foods are also recommended for cholesterol management and their use is growing steadily [5,6]. Among these products, plant sterols and stanols have gained attraction because of their cholesterol-lowering efficacy [7] which has also been acknowledged by authoritative guidelines [8]. Nevertheless, evidence on their long-term safety and efficacy in cardiovascular prevention is lacking, whereas direct access by the public under self-prescription has been criticized and has raised concerns in the scientific society [7,9].

In this context, the aim of the present review is to provide a thorough update on the efficacy and safety of plant sterols/stanol supplementation in the management of dyslipidaemias and cardiovascular prevention.

## 2. Materials and Methods

We searched PubMed, Scopus, and clinicaltrials.gov until January 2023 using the following keywords: plant sterols, plant stanols, cardiovascular disease, cholesterol, supplements, and functional foods. Observational studies randomized clinical trials, systematic reviews, meta-analyses, and committees’ guidelines written in English were considered eligible for the present work. References of eligible articles were scrutinized in case relevant articles were not detected upon initial search.

## 3. Dietary Sources of Intake

The main food sources of plant sterols are vegetable oils, nuts, spreadable fats, breads, cereals, and vegetables [10,11,12]. These contribute by 50–80% of the daily intake of plant sterols, with fruits accounting for a further 12% [10,11,12].

In the typical Western diet, the average daily intake of plant sterols is estimated to be up to ~300 mg, but it can be as high as 600 mg in vegetarians [10,11,12,13]. Sitosterol and campesterol are the main ingested sterols, contributing to the daily intake of plant sterols by ~60% and ~20%, respectively [10,11,12]. On the contrary, the amounts of plant stanols in the diet are much lower and estimated up to 17–24 mg per day (mainly sitostanol and campestanol) [14]. Cereals, especially wheat and rye, are the richest source of plant stanols [12].

## 4. Biology and Mechanism of Action of Plant Sterols/Stanols

### 4.1. Biology

**Figure 1:**
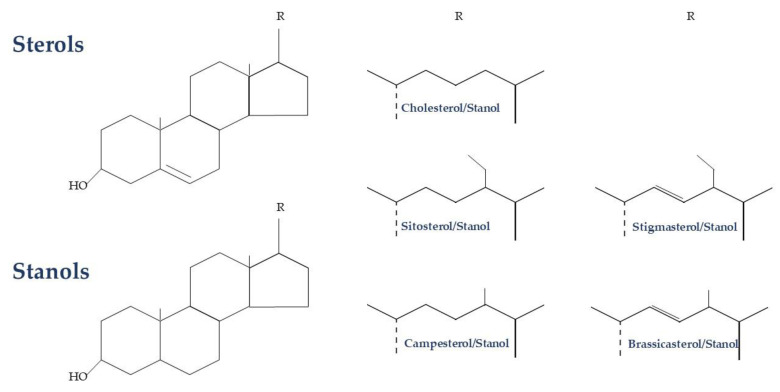
Biochemical structure of plant sterols and stanols.

Plant sterols/stanols are bioactive compounds that have similar functions in mammals to those of cholesterol [7]. Plant sterols are steroidal alkaloids that differ from cholesterol in their side chain at C-24 (methyl or ethyl group) or with an additional double bond in C-22, while plant stanols are 5α-saturated derivatives of plant sterols (Figure 1) [7].

### 4.2. Transport and Circulation

The rate of intestinal absorption of plant sterols/stanols is low (0.05–0.2% for sterols and 0.04–0.2% for stanols) [14,15]. Due to low absorption and increased biliary excretion, the concentration of plant sterols/stanols in the systemic circulation is very low (0.3–1 and 0.002–0.012 mg/dL, respectively) [16]. These levels are 500 and 10,000 times, respectively, lower than those of the circulating cholesterol. Of note, the amount of circulating phytosterols varies in the analytical settings. According to a large meta-analysis of available observational studies investigating the association of circulating phytosterols with CVD, the variation in the concentration of circulating phytosterols was high; the midpoints of the first and third phytosterol tertiles were 1.3 mg/dL and 3.8 mg/dL, respectively [17]. Therefore, any comparison and reference to circulating phytosterols concentration should be made with caution. 

The quantitative distribution of plant sterols/stanols in the various lipoproteins is similar to that of cholesterol, with the largest proportion of those (65–70%) found in the systemic circulation mainly inside LDL particles [7]. There are insufficient data on the concentration of plant sterols/stanols in the various tissues. The tissue concentration in healthy individuals is similar to that of cholesterol; the ratio of cholesterol/phytosterols in the various tissues, ranging from 0.001 to 0.01 × 10^−4^, is similar or lower compared to that in plasma, and the concentration of plant sterols/stanols varies in the various tissues [7].

### 4.3. Mechanism of Cholesterol Absorption Inhibition

The process of uptake of sterols and stanols from the diet until their transport into the systemic circulation consists of three phases. The first phase involves the incorporation of the nonwater-soluble sterols/stanols into the micelles which is necessary for their transport to the apical membrane of the enterocytes [14,18]. This solubilization step is crucial for the transport of sterols/stanols inside the enterocytes and subsequent secretion into the systemic circulation [14,18]. The second phase involves the entry of cholesterol and other sterols/stanols into the enterocyte via a transporter protein called Niemann–Pick C1-Like 1 (NPC1L1) [19]. Inside the enterocytes, sterols are subjected to various processes depending on their chemical structure that constitute the third phase of intestinal administration of sterols/stanols [20,21]. Specifically, cholesterol is mostly esterified through the enzyme acetyl-CoA acetyltransferase 2 (ACAT2) [20,21]. On the contrary, a very small proportion of plant sterols follow a similar pathway, with the majority returning to the intestinal lumen via the ABCG5/ABCG8 transporters [22]. Cholesterol esters, and a much smaller proportion of plant sterols, are incorporated into the chylomicrons which enter the lymphatic system and, later, the systemic circulation [14].

The main putative mechanism of cholesterol reduction through the consumption of plant sterols/stanols is the inhibition of cholesterol absorption from the intestinal lumen through competition for the first phase, i.e., the solubilization [23]. This theory was initially based on an animal study showing that the intragastric administration of a single emulsified lipid meal containing 25 mg of either sitosterol or fucosterol resulted in lower lymphatic cholesterol absorption in comparison to the administration of 25 mg [3H]cholesterol (41% vs. 57%, respectively) [24]. Considering the mean daily consumption of fat (44–78 g) and the approximately 100-fold less intake of total sterols it would be difficult to explain the inhibitory effect of phytosterols with the solubility hypothesis alone. Indeed, plant sterols/stanols have been found to directly interact with other pathways involved in cholesterol homeostasis and mostly with the faecal-neutral sterol excretion known as transintestinal cholesterol efflux (TICE) [25,26,27,28]. Specifically, after incorporating into the intestinal epithelial cells via NPC1L1, plant sterols prevent cholesteryl esters (CE) translocation and esterification by suppressing annexin 2/caveolin 1 (ANXA2–CAV1) and ACAT2, respectively [29]. Phytosterols prevent de novo synthesis of cholesterol by downregulating hydroxymethylglutaryl-CoA (HMG-CoA) reductase and sterol C24-reductase gene expression in the intestine, as well as CE from incorporating into chylomicrons by suppressing microsomal TG transfer protein (MTP) [29]. In the hepatocyte, plant sterols upregulate bile acid synthesis and escalate cholesterol flux through the biliary excretion pathway, whereas the downregulation of the MTP hepatic expression results in the decreased secretion of very-low-density lipoproteins (VLDL) [29]. The upregulation of LDL receptor/LDL-related protein (LDLr/Lrp) by plant sterols also increases cholesterol excretion through the nonbiliary TICE pathway via the ABCG5/G8 and other unknown transporters [29]. Finally, it has been also suggested that phytosterols disrupt cholesterol homeostasis by affecting sterol regulatory element-binding protein (SREBP)-2 processing and liver X receptor (LXR) regulatory pathways [25].

## 5. Effect of Plant Sterols/Stanols on CVD Risk Factors

**Figure 2:**
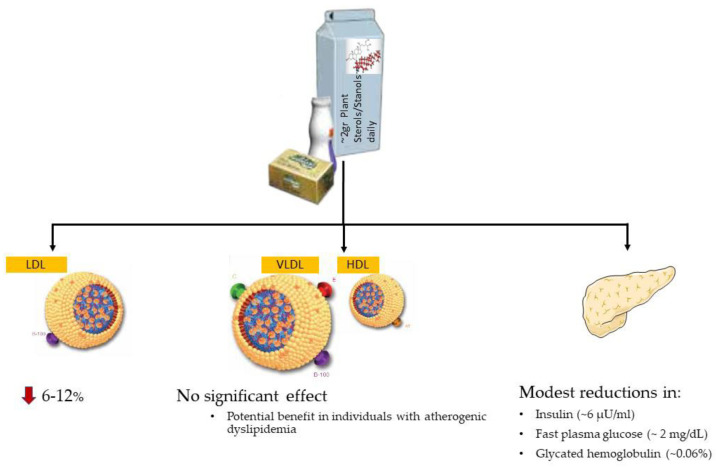
Effect of phytosterol/phytostanol enriched foods on lipids and glucose homeostasis. LDL, low-density lipoprotein; VLDL, very low-density lipoprotein; HDL, high-density lipoprotein; B100, Apolipoprotein B100; C, Apolipoprotein C; E, Apolipoprotein E; A1, Apolipoprotein A1.

Accumulating evidence has established the cholesterol-lowering effect of plant-sterol supplementation (~2 gr daily consumption) and also a potential beneficial impact on other lipoproteins and glucose homeostasis has been described (Figure 2).

### 5.1. Effect on Cholesterol

There is conflicting data regarding the effect of the plant sterols/stanols from natural dietary sources on LDL-C levels. Large epidemiological studies have shown that the dietary intake of plant sterols/stanols is inversely related to LDL-C levels [10,30]. Others have demonstrated that a higher intake of plant sterols/stanols (449 mg/2000 kcal) had the same effect on healthy individuals’ cholesterol levels compared to those consuming smaller amounts (126 mg/2000 kcal) [31]. Even if increasing the consumption of plant sterols/stanols from natural sources has a mild cholesterol-lowering effect, excluding plant sterols/stanols from the diet has been linked to an increase in LDL-C [32].

In contrast to the consumption of plant sterols/stanols from natural dietary sources, studies have shown that the fortification of foods with plant sterols/stanols is associated with effective LDL-C reduction [33]. The LDL-C lowering effect of plant sterols/stanol supplementation has been confirmed in several meta-analyses, including a large number of randomized, placebo-controlled clinical trials which investigated the relationship between the dose of plants sterols/stanols and their efficacy, as well as the impact of the food format of phytosterols [34,35,36,37]. A recent meta-analysis of 124 randomized placebo-controlled trials including 9600 adults showed that the average intake of plant sterols/stanols was 2.1 g/day (range 0.2–9.0 g/day) and, overall, a consistent dose–response relationship for LDL-C lowering by 6–12% with intakes of 0.6–3.3 g/day was found [37]. In children, studies have shown that consumption of foods enriched with plant sterols/stanols (1.5–3.0 g daily) reduces LDL-C levels by 5–19% [7]. Supplementation with phytosterols and phytostanols lowers LDL-C regardless of the baseline levels, as is the case of individuals with familial hypercholesterolaemia (FH) [38]. According to a recent meta-analysis, the combination of plant-stanol supplementation with a cholesterol-lowering diet decreased total cholesterol (mean difference, MD: −0.62 mmol/L, 95% confidence intervals, CI: −1.13, −0.11) and LDL-C (MD: −0.58 mmol/L, 95% CI: −1.08, −0.09) in FH individuals when compared with a cholesterol-lowering diet alone [38]. The same was noted for the supplementation with plant sterols (MD: −0.46 mmol/L, 95% CI: −0.76, −0.17, for cholesterol and MD: −0.45 mmol/L, 95% CI: −0.74, −0.16, for LDL-C) [38].

At intakes higher than 3 g/day, there is a tapering-off effect for the LDL-C-lowering effect of phytosterols and phytostanols as the inhibition of cholesterol absorption seems to be a saturable process [39]. However, due to the limited number of clinical trials with intakes >4 g/day, it remains speculative whether the dose–response relationship would continue and whether efficacy would differ between phytosterols and phytostanols at higher intakes [40]. On the other hand, both plant sterols and stanols have a similar LDL-C lowering effect at intakes ≤ 3.3 g/day with no safety issues noted [40]. 

Phytosterols and phytostanols supplementations are effective in various types of food formats such as fat-based foods such as margarines, spreads, and dairy-type foods (yoghurt, milk), as well as in dietary supplements including capsules and tablets [36,41]. There is no difference in the efficacy between free and esterified plant sterols/stanols [36,41], nor does the choice of dietary fatty acid used for esterification or the source of plant sterols/stanols seem to have a meaningful impact on LDL-C lowering efficacy [42]. In comparison with multiple daily intakes, a once-a-day intake, especially with a light breakfast in the morning, seems less potent in lowering LDL-C [36,41,43]. The consumption of plant sterols/stanols with a meal, instead of fasting conditions, along with the fat content of a meal, are critical factors for the LDL-C efficacy of enriched foods and supplements with plant sterols/stanols [44].

A single-centre, prospective, randomized, single-blind clinical trial including 190 adults at high CVD risk with hypercholesterolaemia (LDL-C 70–189 mg/dL) recently compared the lipid-lowering and anti-inflammatory effects of a low-dose statin (i.e., rosuvastatin 5 mg) with a placebo and six common supplements, including plant sterols/stanols [45]. A higher LDL-C reduction was noted in patients treated with rosuvastatin 5 mg (*n* = 25) compared with those taking 1.6 g plant sterols (*n* = 25) (MD: 33.5%, 95% CI: 27.4, 39.6, *p* < 0.001). Of note, the supplementation with plant sterols had no effect on participants’ LDL-C levels (MD: −1.7%, 95% CI: −7.9, 4.4) [45]. 

### 5.2. Effect on Other Lipid Fractions

In contrast to hypercholesterolaemia, few studies have investigated the effect of plant sterol/stanol supplementation in patients with atherogenic dyslipidaemia. Sparse evidence have suggested that a daily consumption of 1.5–2 g/day of plant sterols/stanols reduces triglycerides by 6–20% and increase high-density lipoprotein cholesterol (HDL-C) by 5–11%, but mainly in individuals with atherogenic dyslipidemia [46]. Nevertheless, a recent network meta-analysis of 131 trials on the comparative effect of nutraceuticals on the lipid profiles of 13,062 adults has not shown any significant effect of plant sterol/stanol supplementation either on triglycerides (MD: −0.11 mmol/L, 95% CI: −0.28, 0.06) or on HDL-C (MD: 0.04 mmol/L, 95% CI: −0.03, 011) [47].

Regarding apolipoproteins, studies have shown that the intake of foods enriched with plant sterols/stanols reduces apolipoprotein B, but does not affect the levels of apolipoprotein A-I [46]. Regarding lipoprotein (a), a meta-analysis including 7 studies with 363 participants has recently revealed a statistically, but not clinically significant, reduction following phytosterol supplementation (MD: −0.025 mg/dl, 95% CI: −0.045, −0.004) [48].

### 5.3. Effect on Glucose Metabolism

Over the last decade, emerging evidence supports the theory that supplementation with phytosterols and phytostanols has a hypoglycemic effect. Experimental studies have shown that oral administration of ß-sitosterol in hyperglycemic rats decreased their glucose and insulin levels [49]. The proposed pathophysiological mechanism involves the mediation of the uptake of glucose by the adenine monophosphate-activated protein kinase (AMPK) [50]. Randomized trials in humans have confirmed the beneficial, but not clinically significant effect of food enrichment with plant sterols/stanols on glucose metabolism. Specifically, a recent meta-analysis including 20 randomized clinical trials with 1306 adults has shown that the supplementation with phytosterols decreases insulin levels (MD: −6.426 μU/mL, 95% CI: −7.187, −5.665) [51]. Moreover, significant changes in fasting plasma glucose levels (MD: −1.942 mg/dL, 95% CI: −3.637, −0.246) and glycated hemoglobulin (MD: −0.059%, 95% CI: −0.114, −0.004) were recorded in those receiving 1–2 g of phytosterols [51]. 

## 6. Effect of Plant Sterols/Stanols on Atherosclerosis and CVD Clinical Outcomes

### 6.1. Effect of Plant Sterols/Stanols on Atherosclerosis in Animal Studies

Animal studies have demonstrated controversial results regarding the effect of diet supplementation with phytosterols on atherosclerosis (Table 1). 

Phytosterol administration (1–2% wt/wt) reduced the formation or retarded the progression of atherosclerotic lesions in apolipoprotein E deficient mice [52,53,54] and improved aortic function in hamsters fed with a high-cholesterol diet [55]. In these studies, they used phytosterol mixtures containing 69% β-sitosterol, 16–87.6% sitostanol, 15% campesterol, and 9.5–30.1% campestanol [52,53,54,55]. On the other hand, a diet supplemented with 2% phytosterols impaired endothelium-dependent vasodilation and increased cerebral lesion size after middle-cerebral artery occlusion in apolipoprotein E −/− rodents [56]. Of note, sterol supplementation was designed to reflect the composition of commercially available spreads in that study (46.2% sitosterol, 2.3% sitostanol, 25.3% campesterol, 0.6% campestanol, 19.1% stigmasterol, 1.2% brassicasterol, and 4.9% other plant sterols) [56]. It should be mentioned that in this animal model, plasma cholesterol concentration did not change during treatment, whereas the dose consumed by mice in this experiment, when expressed per kilogram of body weight, was about 100-fold higher compared with the typical human consumption. After comparing ezetimibe with plant sterols, several-fold increased plasma levels of plant sterols and two- to four-fold larger atherosclerotic plaques were observed in mice fed the plant sterols [56]. Furthermore, the plasma concentration of plant sterols was strongly correlated with atherosclerotic lesions [56]. Of note, the results of this study should be interpreted under the scope of the comparison between an effective cholesterol-lowering drug and a dietary intervention with phytosterol supplementation, as well as the additional pleiotropic effects of ezetimibe that are unrelated to cholesterol reduction and might be involved in the potentiation of atherosclerotic plaque regression [77]. Although little is known about the atherogenicity of oxidized plant sterols (oxyphytosterols), another study showed that the supplementation with oxyphytosterols (0.025% wt/wt) increased the proportion of severe atherosclerotic lesions in LDL receptor-deficient (LDLR+/−) mice [78].

### 6.2. Effect of Plant Sterols/Stanols on Atherosclerosis in Human Studies

There is insufficient data regarding the effect of plant sterol/stanol supplementation on the human endothelium (Table 1). Although one study showed that a daily supplementation with 1.93–1.98 g plant sterols/stanols reduced the diameter of the brachial artery [57], others have shown a neutral effect with a similar daily dose (2 g/d) on other markers of endothelial function, such as flow-mediated dilatation and carotid intima media thickness [58,79]. Of note, the short follow-up, and the inclusion of subjects with normal endothelial function, were limitations of these studies.

Other studies have shown no benefit of plant sterol/stanol supplementation on coagulation and platelet adhesion, as well as various markers of oxidative stress and inflammation [59,60,61].

### 6.3. Effect of Plant Sterols/Stanols on Cardiovascular Events 

#### 6.3.1. Randomized Trials

To date, there are no randomized placebo-controlled trials to investigate the impact of phytosterol supplementation on hard cardiovascular outcomes (Table 1). Cardiovascular outcome trials investigating foods fortified with plant sterols/stanols in individuals at low or moderate CVD risk are not feasible, given the sample size (*n* > 50,000) required for adequate statistical power [2,7]. Moreover, it would be difficult to demonstrate the unequivocal, but relatively small, additional LDL-C lowering effect of plant sterol/stanol supplementation in a clinical trial of optimally treated high cardiovascular-risk patients, even in the case of 25–30,000 participants [2,7]. On the contrary, a few studies have investigated the association of circulating plant sterols/stanols and related diseases with CVD.

#### 6.3.2. Observational Studies

Sitosterolaemia or phytosterolaemia is a rare autosomal recessive lipid disorder characterized by premature CVD and markedly elevated plant-sterol concentrations in the blood due to increased intestinal absorption and decreased biliary secretion [62,63]. This happens due to homozygous or compound heterozygous loss-of-function mutations in ABCG5 and ABCG8 genes which normally limit the intestinal absorption of plant sterols/stanols [62,63]. The prevalence of homozygous/compound heterozygous sitosterolaemia in the general population is estimated up to 1 in 200,000 and serum sitosterol concentrations are usually >1 mg/dL (10 μg/mL) in these patients [63]. Although plasma total cholesterol and LDL-C levels are usually normal or modestly elevated [14], severe hypercholesterolaemia can also be noticed [63,80]. Patients with sitosterolaemia usually present with cutaneous or tendon xanthomas and mostly with premature atherosclerotic CVD, but they may also manifest with macrothrombocytopaenia leading to severe bleeding episodes as well as splenomegaly and haemolytic anaemia [63,81].

Apart from the rare cases of sitosterolaemia, the association between circulating levels of plant sterols/stanols and CVD risk in the general population has also been investigated (Table 1). A few studies have linked high concentrations of circulating phytosterols with prevalent CVD, but others have found a neutral effect [17,64,65,66,67,68,69,70,72,73]. Most recent data indicate a positive association with increased cholesterol absorption, as expressed by the campesterol/cholesterol ratio, with the risk of in-stent restenosis in 59 patients with stable coronary artery disease [71]. Another cross-sectional study has recently shown a positive association of carotid intima-media thickness with serum campesterol concentrations (odds ratio, OR: 1.71, 95% CI: 1.04, 2.82, *p* < 0.05) and an inverse association between both lathosterol/campesterol (OR: 0.29, 95% CI: 0.11, 1.80, *p* < 0.05) and lathosterol/sitosterol (OR: 0.45, 95% CI: 0.22, 0.95, *p* < 0.05) ratios in 270 asymptomatic individuals [82]. For the interpretation of these results, one should keep in mind that campesterol and the investigated ratios are markers of cholesterol absorption efficiency. In addition, there are certain limitations that should be considered in all past studies investigating the association between circulating phytosterols and CVD risk, such as differences in sample size, design, the source and the cause of elevated circulating phytosterols, the methods of their measurement in plasma, as well as lack of adjustment for confounding factors, prevalence of other cardiometabolic diseases, the ABCG5/8 phenotype, and the dietary intake of plant sterols. A large meta-analysis including 17 studies (cohorts, parallel- or cross-over trials, and cross-sectional/case–control/nested case–control studies) with 11,182 individuals demonstrated no significant association of circulating campesterol (relative risk, RR: 1.02, 95% CI: 0.94–1.09) and sitosterol (RR: 1.06, 95% CI: 0.84–1.34) with the presence of CVD, although the possibility of an accumulation of plant sterols/stanols in vascular cells due to the increase in their circulating concentrations could not be excluded [17]. Among the included studies, plant-sterol concentrations varied substantially; sitosterol concentration ranged 0.15–0.35 mg/dL, and campesterol 0.25–0.64 mg/dL, respectively [17]. Of note, observational cohorts do not provide a causality and are susceptible to several confounding factors that might have not been considered in their analysis.

#### 6.3.3. Genetic Studies

Genetic studies have demonstrated that mutations leading to increased intestinal absorption of plant sterols/stanols are associated with increased CVD risk (Table 1). According to a large meta-analysis of 11 different studies comprising over 13,000 cases with CAD and controls, ABCG8 and ABO alleles associated with elevated circulating phytosterol levels displayed significant associations with the presence of CAD (rs4245791, OR: 1.10, 95% CI: 1.06, 1.14; rs657152, OR: 1.13, 95% CI: 1.07, 1.19) [75]. On the other hand, ABCG8 associated with reduced circulating phytosterol levels were associated with reduced risk of CAD presence (rs41360247, OR: 0.84, 95% CI: 0.78, 0.91) [75]. Despite the advantages of genome-wide association studies, they do not necessarily establish a causal relationship. Of relevance, circulating phytosterols are used to measure cholesterol intestinal absorption efficiency [83]. In addition, the investigated risk alleles in ABCG8 and ABO have been found to correlate not only with higher circulating phytosterol concentration but also with total and LDL-C levels [84,85]. Hence, the association between these variants and the presence of CAD could be attributed to the atherogenic effect of the elevated cholesterol and LDL-C levels in high cholesterol absorbers. Another genome-wide study, including sequence data of 91,002 participants, showed that carriers of NPC1L1 inactivating mutations, resulting in LDL-C reduction by ~12 mg/dL, were associated with a 53% relative CAD risk reduction [74]. Based on this evidence, the genetic inhibition of NPC1L1 was speculated to lower CVD risk by also reducing phytosterol absorption [74]. A recent study examined the effects of ABCG5/8 and NPC1L1 variants on nonhigh-density lipoprotein cholesterol (non-HDL-C) (*n* = 610,532) and circulating phytosterol levels (*n* = 3039), as well as CAD risk in individuals of European origin from Iceland, Denmark, and the UK Biobank (105,490 cases and 844,025 controls) [76]. Nine rare ABCG5/8 coding variants with substantial impact on non-HDL-C and circulating phytosterols were found; stigmasterol concentration was 2.85 and 2.68 standard deviation units higher than the mean in homozygous or compound heterozygous carriers [76]. Carriers of these variants were at increased risk of CAD [76]. A genetic-risk score of ABCG5/8 variants predicting 1 mmol/L increase in non-HDL-C was associated with a two-fold increase in CAD risk (OR: 2.01, 95% CI: 1.75, 2.31) compared with a 54% increase in CAD risk (OR: 1.54, 95% CI: 1.49, 1.59) associated with a score of other variants in genes expect for ABCG5/8 (such as apolipoprotein B, 3-hydroxy-3-methyl-glutaryl-coenzyme A reductase -reductase, proprotein convertase subtilisin/kexin type 9, and LDL receptor) predicting the same increase in non-HDL-C [76]. Based on these results the authors suggested that non-HDL-C could only explain around 62% of the CAD risk inferred from the effect of variants in the genomic risk score of ABCG5/8, whereas the remaining 38% should be attributed to other mechanisms [76]. Considering the relationship between ABCG5/G8 variants and circulating phytosterol levels, elevated circulating phytosterol levels might be a plausible explanation for the excess CAD risk [26]. On the other hand, these findings should be interpreted under the scope of certain limitations [86]. First, the association of the investigated genotypes with plasma cholesterol and phytosterol levels were expressed as per standard deviation units instead of absolute serum concentrations. Only by showing the differences in absolute circulating phytosterol levels between the genotypes, it would be possible to interpret the clinical relevance of the 20–30% increase in serum phytosterol concentrations caused by the consumption of foods enriched with phytosterols [7]. Of note, circulating phytosterol levels far exceed the normal range in individuals with sitosterolaemia who are frequently diagnosed with premature atherosclerotic CVD [62,63,87]. Furthermore, the authors focused only on campesterol, sitosterol, and stigmasterol. Considering that serum cholestanol is used to measure intestinal cholesterol absorption efficiency, if the relevant data were available and showed that cholestanol resulted in the same conclusion, it would be possible that the excess CAD risk noted in that study could be related to cholesterol absorption itself and not to the circulating phytosterols. 

## 7. Clinical Implications and Practical Issues

### 7.1. Official Scientific Guidelines

The most recent guidelines of the European Society of Cardiology/European Atherosclerosis Society, endorse the use of functional foods enriched with plant sterols/stanols (≥2 g/day with the main meal) (i) in individuals with high cholesterol levels at intermediate or low CVD risk who do not qualify for pharmacotherapy, (ii) as an adjunct to pharmacological therapy in high- and very-high-risk patients who fail to achieve LDL-C goals on statins or are statin intolerant, and (iii) in adults and children (aged > 6 years) with familial hypercholesterolaemia [2]. Of note, these guidelines were published before the conduct of the genetic studies linking genetic susceptibility to the high absorption of certain phytosterols (i.e., sitosterol, campesterol, stigmasterol, and brassicasterol) with the risk of CAD and, thus, raising concerns about the safety of phytosterols [74,75,76]. Considering the fact that food supplementation includes a mixture of various phytosterol/stanols at different concentrations [88] and the limitations of these studies, the harmful effects of phytosterol supplementation cannot be concluded yet. Ultimately, future studies are needed to further investigate whether the relatively small increase in the plasma concentration of circulating phytosterols induced by dietary supplementation attenuates atherosclerosis and increases CVD risk. Until then, physicians should speculate on the controversial evidence on the association between plant-sterol supplementation and atherosclerosis as well as its modest cholesterol-lowering potency. In this regard, consumption of foods enriched with plant sterols/stanols can be recommended in individuals with hypercholesterolaemia not qualifying for lipid-lowering therapy, where their cholesterol-lowering effect and the associated cardiovascular benefit is expected to exceed any adverse risks. Physicians should also remain vigilant about overconsumption and remind their patients that phytosterols should not replace lipid-lowering drugs.

It is important to note though that phytosterol supplementation is contraindicated in the rare case of patients with sitosterolaemia [2,63]. Although familial sitosterolaemia is traditionally considered as a recessive disorder, increased sitosterol and LDL-C levels, along with elevated CAD risk, have been also described in heterozygous carriers of a loss-of-function variant in ABCG5/8 [89]. Thus, this evidence also raises concerns for the latter group for whom phytrosterols/phytostanols supplementation could lead to adverse events.

### 7.2. Data on Consumption of Foods Enriched with Plant Sterols/Stanols

Data on phytosterol consumption in the community mainly derives from studies conducted in the past decades aiming to assess potential risks deriving from the overconsumption of foods with added plant sterols and stanols. A large postlaunch monitoring (PLM) survey, conducted in 2011 in five European countries (the Netherlands, Belgium, UK, France, and Germany), collected data on consumer purchases of foods with added plant sterols using large household panels (5000–30,0000 per country, 91,000 in total) [90]. Households being consumers of such products constituted 12.7% of the population [90], in accordance with earlier estimations reported by EFSA (10–15%) [91]. However, the consumption of plant sterols/stanols is not the same for all family members, i.e., children. The PLM survey reported a small proportion (2.5–6.4%) of households with children under 5 years buying enriched foods with plant sterols; children consumed a much smaller total volume of these products [90]. Although the proportion of children consuming these products is generally estimated to be low, sparse evidence supports the opposite; for instance, 21% of preschool children were characterized as consumers in a Belgian survey [92]. Interestingly, the 2011 PLM survey estimated the average intake of plant sterols to be 0.35–0.86 g/day (range among countries), while the Netherlands presented a higher intake of >3 g/day [90]. Even though average intake has been reported to be somehow greater in other studies [91,93], overall data show that overconsumption is rather unlikely. 

### 7.3. Safety Issues and Concerns 

Data on the safety of plant-sterol supplementation derived from long-term clinical trials do not exist [7]. However, evidence from postlaunch monitoring has raised no major concerns about the overconsumption of foods with added plant sterols [7,90]. In addition to the potential proatherogenic effects, there are other safety concerns regarding phytosterol consumption. First, a recurring observation in a few studies concerns the interference of phytosterols with the absorption of fat-soluble vitamins and mostly for the highly lipophilic hydrocarbon carotenoids (beta-carotene, alpha-carotene, and lycopene), probably via the suppression of intestinal absorption [94,95]. However, any decline in fat-soluble vitamin levels induced by plant sterol/stanol intakes can be mediated by increasing the consumption of fruits and vegetables [94,95]. Although cancer was among the initial areas of concern surrounding phytosterol consumption [7], experimental models and observational studies support a potential protective role of plant sterols/stanols against certain types of cancer [95,96,97]. Indeed, an ongoing randomized trial with a cross-over design (plant sterol intervention for cancer prevention, PINC) will test whether phytosterols alter the ability of noncancer cells (adipocytes, fibroblasts, and macrophages) collected from hypercholesterolemic volunteers to change chemotherapy response and metastatic process in breast cancer cells [98].

As far as the safety of coadministration of plant sterols/stanols with other lipid-lowering drugs is concerned, the additional ~10% LDL-C reduction appears to be maintained with statins, ezetimibe, and fibrates [79]. However, there is limited evidence about bile acid sequestrants and the coadministration with plant sterols is not recommended as they reduce their absorption [79]. In any case, maximum synergistic effects can be achieved if plant sterols are coadministrated with drugs or other supplements that target different mechanisms of LDL-C lowering than cholesterol absorption.

## 8. Conclusions

Current guidelines recommend plant sterols and plant stanols in the amount of approximately 2 g/day with the goal of reducing LDL-C by approximately 10% in combination with dietary changes. Despite their undoubtable cholesterol-lowering effect, past experimental and human studies have demonstrated controversial results regarding the effect of phytosterol consumption on various markers of atherosclerosis. Compelling evidence from recent genetic studies have linked elevated plasma concentrations of circulating plant sterols with CVD presence, thus raising concerns about the safety of phytosterol supplementation. Considering the lack of, and the inability to design, randomized clinical trials addressing cardiovascular efficacy and safety, future studies could clarify whether the relatively small increase in the plasma concentration of circulating phytosterols induced by phytosterol-enriched food attenuates atherosclerosis and increases CVD risk. In the meantime, both physicians and consumers should keep in mind that this recommendation might be applied to individuals with hypercholesterolaemia who are expected to have a cardiovascular benefit, but also that the daily intake must not exceed the recommended dose. Finally, plant sterols/stanols should not be viewed as a substitute for much more effective and evidence-based cholesterol-lowering therapies, especially in individuals at high CVD risk.

## Figures and Tables

**Table 1:** Effect of plant sterols on atherosclerosis and cardiovascular disease.

Study Design	Findings
Animal studies	Phytosterol administration reduced the formation of new atherosclerotic lesions in apolipoprotein E −/− mice [52,53] Phytosterol administration retarded plaque formation in apolipoprotein E −/− mice [54] Phytosterol administration improved aortic function in hamsters fed with a high-cholesterol diet [55] Diet supplemented with phytosterols impaired endothelium-dependent vasodilation in apolipoprotein E −/− mice [56] Phytosterol administration increased cerebral lesion size after middle-cerebral artery occlusion in apolipoprotein E −/− mice [56] Larger atherosclerotic plaques in mice fed with plant sterols compared to ezetimibe [56] Levels of circulating phytosterols were associated with the progression of atherosclerosis [56]
Human observational studies	Phytosterol consumption reduced brachial artery diameter in patients with hypercholesterolesterolemia [57] Diet supplementation with phytosterols had no effect on flow-mediated dilation [58] Phytosterol consumption had no benefit on coagulation, platelet adhesion, oxidative stress, and inflammation [59,60,61] Patients with sitosterolaemia have extremely elevated levels of circulating phytosterols and present with premature atherosclerotic CVD [62,63] Some epidemiological and case–control studies show an association between circulating phytosterols and increased CVD risk [64,65,66,67,68,69,70,71] Other observational studies report no correlation or decreased CVD risk with increased plasma concentrations of phytosterols [72,73] The largest meta-analysis including 17 observational studies (*n* = 11,182) demonstrated no significant association of circulating campesterol with CVD [17]
Human genetic studies	NPC1L1 inactivating mutations were associated with a 53% relative CAD risk reduction [74] ABCG8 and ABO alleles associated with elevated circulating phytosterol levels displayed significant associations with the presence of CAD [75] A genetic-risk score of ABCG5/8 variants predicting 1 mmol/L increase in non-HDL-C was associated with a two-fold increase in CAD risk compared with a 54% increase in CAD risk associated with a score of other non-ABCG5/8 variants [76]
Randomized trials	To date, there are no randomized placebo-controlled trials to investigate the impact of phytosterol supplementation on major cardiovascular events

CVD, cardiovascular disease; CAD, coronary artery disease; ABCG5/8, ATP-binding cassette transporters G5 and G8; NPC1L1, Niemann-Pick C1-Like; ABO, blood group; non-HDL-C, non-high-density lipoprotein cholesterol.
